# Middlesex Hospital

**Published:** 1893-11-18

**Authors:** 


### The Institutional Workshop.

### WITHIN THE HOSPITALS.

### MIDDLESEX HOSPITAL.

(By Our Special Commissioner.)

This institution, instituted in 1745, is one of the best examples of the H plan of hospital construction to be found in the country. Those who are interested in hospital construction should visit Middlesex and then go to the Radcliffe Infirmary, Oxford, so as to gain a practical insight into the evils and costliness which result from an ill-considered departure from the original plan, like that which has taken place at Oxford. Middlesex still remains true to the H plan, and so illustrates the adaptability and soundness of the principles it embodies, both from an administrative and hygienic point of view. It is true that the lower wards on one side have less cross ventilation than those on the upper floors, but this has nothing whatever to do with the plan as such. Middlesex contains the best system of ventilation by ward windows, for which it has long been famed. We were sorry to see that the sisters still sleep in rooms attached to the wards, as we had hoped so excellently managed a nursing department as that at Middlesex would have been strong enough to have eradicated an evil which ought to be confined to hospitals of an inferior type. The nursing at Middlesex is very good, and Miss Thorold, who has been there for upwards of a quarter of a century, is still one of the most active and zealous of matrons. Indeed, the nursing department is so important historically, and so good, that we propose to devote a separate article to it.

#### Business and Results.

We rejoice to find in the course of our inspections that evidence accumulates that committees and governors are becoming more and more alive to the importance of introducing sound business principles into the management of our hospitals. Nowadays the number of patients to be handled, and the resultant expenditure, are so large as to necessitate the application of the best methods of business to the conduct of the affairs of a voluntary hospital. We have long insisted that no important hospital should be placed in charge of a Secretary-Superintendent who has not had previous training in a recognised institution of importance. It is, in our view, too great a risk to select a gentleman for such a post who has no knowledge whatever of hospital affairs. Our inspections show how much a hospital gains in income, in reputation, and efficiency, by having at its head a trained and experienced man of business who has made a study of hospital affairs. The Middlesex Hospital is especially fortunate in this respect. It has a principal medical officer, Mr. Farden, who has devoted very many years of his life to the work,, and whose services, we are glad to say, the governors handsomely recognised on a recent occasion. Mr. Farden is warmly interested in the hospital, and the value of his work is known and appreciated outside. This is proved by the fact that he was recently selected to carry out an investigation at Dewsbury at the instance of the Royal Commission on Vaccination, in conjunction with that most able pathologist, Dr. Coupland. Miss Thorold, as we have said, has been in charge of Middlesex for nearly a quarter of a century. She has devoted her life to the work, and is to-day as enthusiastic and energetic as any matron in the world. Miss Thorold's great strength lies in the fact that she makes it her business to attend personally to everything, and has so avoided the evil tendencies of the present day which have led more than one matron in charge of a large hospital to depend upon eyes and ears other than her own, a system which is apt to bring upon herself and upon her institution difficulties which would otherwise most certainly be avoided.

Mr. Melhado, Secretary-Superintendent, is one of the most experienced and able hospital officials of the day. He has studied hospital administration in all its branches with success. He is enthusiastic in his work, and his energy and persistence are worthy of all praise. So much is this the case, that the governors have not only sanctioned his marriage, a step which in Mr. Custance's days led to a change in the Superintendent, but they are about to build him a house, so that he may be able to devote his whole energies under the most favourable circumstances to the work of the hospital. He has also recently received a handsome addition to his emoluments. We mention these matters, not only in justice to Mr. Melhado, but to encourage other hospital committees to take the first favourable opportunity of enforcing business methods, and so to secure a wider support and a closer efficiency in the administration of the affairs of the institutions for which they are responsible.

#### Recent Improvements.

A great many improvements have been made at Middlesex Hospital during the last few years, under Lord Sandhurst and Mr. Melhado. Not the least of these has been the introduction of the electric light at a cost of about £2,000, which has not only added to the comfort of the patients, but has immeasurably increased the facilities for clinical work. The wards have been redecorated throughout; the floors have been laid down in teak, and teak fittings have been added to the ward kitchens. Teale's grates have been introduced, and lend a most attractive appearance to the wards. The fittings are on the whole good, and the appearance of the wards could hardly be improved. We have no doubt that many of the sisters would be glad if some of the more wealthy residents in the district would bear Middlesex in mind, and send from time to time fresh flowers, both cut and in pots. The recent structural alterations have led to a surprising innovation. Heretofore it has been laid down that "cleanliness is next to godliness," but we imagine, that without consulting the medical staff, someone has decided that all the baths shall be moved to the basement. It is true that one portable bath is left on each floor; but a portable bath is a very great trouble, and at best but a make-shift. The baths in the basement are three in number, and we hope that a similar plan will not be followed by any other hospital, as we are confident it is not fair to either the nurses or the patients, and cannot make for efficiency or cleanliness. It is fair to add that the really splendid condition of the children in the children's wards proves that the nurses are superior to the absence of the usual bathing facilities which have so strangely been denied them of late. With this exception, taking Middlesex as a whole, the recent alterations have proved a decided success, and we congratulate the authorities upon their completion. This success is especially apparent in the new drainage arrangements. The new mortuary is also excellent in many respects. The approaches to it, too, have been well thought out and cleverly contrived. The new chapel is a graceful addition to the institution, and the beauty of the marble selected for the interior decoration is very striking. The limited site has dwarfed the width of the aisle, and so partly destroyed the general effect, which would otherwise have been charming. The new chapel will, we hope, add much to the comfort and well-being of the inmates in many ways.

The new Residential College, in which all the resident medical officers now reside, must be a boon to them, and to the students of Middlesex also. We commend it to the attention of parents who have sons about to enter the medical profession, and would suggest that they should pay it a visit before selecting a medical school.

### Work Still to be Done.

As the present Board are evidently progressive, we venture to urge them to spend yet more money, and so to make Middlesex Hospital complete in every department. At the present time both the laundry and the kitchens are situated in the basement. This arrangement is hygienically indefensible, and probably dangerous, and ought to be changed. On every floor the smell of the kitchens was painfully apparent, and in these days it is not necessary to waste words in condemning a hospital laundry situated in the basement of a great hospital. It is also desirable, we believe, for further accommodation to be secured for cancer cases, and we would suggest its being provided in a separate wing, the kitchens and laundry being then moved to the top of the hospital, unless arrangements could be made to have the laundry work done elsewhere. At any rate, these improvements are urgently called for, and we shall hope to hear that plans are in contemplation to carry them out with as little delay as possible. Middlesex deserves the support of the public, especially on account of the cancer wards, where many poor sufferers find a comfortable resting place in the last days of their painful lives. We could wish that one or two wealthy people would manifest a warm interest in these cancer wards, and by placing adequate funds at the disposal of the committee enable them to promptly erect a new cancer wing. A visit to the cancer wards, with the view of proving their enormous value to the race, would we believe, lead many people to give large sums to this beneficent work. May we hope that some of the readers of The Hospital will devote an afternoon to their inspection.

